# Impact of play‐based intervention and parental support on motor skills, behavioral problems, and parenting stress in Japanese children with probable developmental coordination disorder: A randomized controlled trial

**DOI:** 10.1002/pcn5.70256

**Published:** 2025-11-24

**Authors:** Ryota Hatanaka, Yumi Higuchi, Yasuko Takahashi, Ayako Hisari, Keiko Sakai, Masatoshi Takeda

**Affiliations:** ^1^ Faculty of Rehabilitation Osaka Kawasaki Rehabilitation University Osaka Japan; ^2^ Graduate School of Rehabilitation Science Osaka Metropolitan University Osaka Japan

**Keywords:** behavioral problems, developmental coordination disorder, intervention, parenting stress, randomized controlled trial

## Abstract

**Aim:**

Developmental coordination disorder (DCD) is associated with motor impairments, behavioral and emotional challenges, and elevated parenting stress. While motor‐skill training is an effective intervention for DCD, its impact on psychosocial effects remains uncertain. Therefore, in this study, we aimed to evaluate the effectiveness of combined motor‐skill training and direct parental support‐based intervention in reducing behavioral problems and parenting stress in Japanese children with probable DCD (pDCD).

**Methods:**

A randomized controlled trial was conducted involving 20 children aged 6–15 years who met the diagnostic criteria for pDCD. Group comparisons were performed using the Mann–Whitney *U* test for non‐normally distributed data. Participants were randomly assigned to either an intervention or a control group (*n* = 10/group). The intervention group received weekly 90‐min sessions over 8 weeks, including task‐oriented motor training, social skills development, and parental support. Primary outcome measures included the Movement Assessment Battery for Children‐Second Edition, Child Behavior Checklist, and the Parenting Stress Index‐Child Domain.

**Results:**

No significant improvements in motor function were observed in either group. However, a significant reduction in both behavioral problems and parenting stress was observed in the intervention group, whereas no statistically significant changes were observed in the control group.

**Conclusion:**

The integrated intervention may help reduce parenting stress and alleviate behavioral challenges in children with pDCD. These results highlight the importance of multidimensional support strategies that address both child and parental needs.

## INTRODUCTION

Developmental coordination disorder (DCD) is a neurodevelopmental condition characterized by difficulties in motor coordination that affect activities of daily living and academic achievement.^1^ DCD occurs when a delay in the development of motor skills or difficulty coordinating movements results in a child being unable to perform common, everyday tasks. It affects approximately 5%–8% of children aged 5–11 years in the United States of America.^1^ The movements of children with DCD are often described as “clumsy” and “uncoordinated” and frequently lead to performance difficulties that most typically developing children can perform easily. It is characterized by significant impairment in executing coordinated motor skills that is inconsistent with the child's chronological age and persists despite opportunities for skill learning and practice being provided. Furthermore, DCD, beyond motor deficits, is associated with an elevated risk of psychosocial problems, which often requires involvement of psychiatrists, neurologists, and other specialists.^1^

In the last few decades, several studies have explored the mechanisms, interventions, and consequences of DCD. However, little has been disseminated regarding clinical and educational practices in some countries, including Japan. A survey of 450 clinical professionals in Germany and the United Kingdom found that only 58% had heard of DCD,^2^ indicating that even in Western countries, awareness and understanding of the disorder remain limited. Furthermore, Gabbard and Tamplain highlighted limitations in DCD‐related screening questionnaires, noting that their effectiveness in evaluating DCD varies depending on the context, expertise of the user, and intended purpose.^3^

According to international clinical practice recommendations,^4^ children with DCD often experience internalizing problems, such as depression and anxiety, and externalizing behavioral problems, including attention‐deficit/hyperactivity disorder (ADHD). A 2020 systematic review of 581 studies reported that anxiety symptoms occur in 17%–34% and depressive symptoms in 9%–15% of individuals with DCD,^5^ highlighting the emotional burden associated with DCD and the importance of addressing internalizing symptoms in clinical practice. Anxiety is associated with elevated risks of suicidal ideation, suicide attempts, and mortality, whereas depression typically manifests as anhedonia and social withdrawal. In a more recent study of medical records from 93 children with DCD referred to a rehabilitation center, behavioral and emotional problems, assessed using the Child Behavior Checklist (CBCL) and Teacher Report Form, were observed in approximately two‐thirds of cases.^6^ Among subtypes, children with general motor coordination impairments were most affected, whereas those with gross motor problems were least affected. These children presented with more problems at home than at school, suggesting that behavioral and emotional challenges impose a substantial burden on parents. In a survey of 174 parents of children with DCD, approximately two‐thirds reported clinically significant levels of parenting stress.^7^ These findings suggest that children with DCD and their parents may experience challenges not only in daily functioning but also in psychosocial domains. Therefore, interventions should address both motor development in children and the emotional well‐being of their caregivers.

Families raising children with DCD, as well as children with DCD themselves, encounter diverse challenges, including motor impairments, psychosocial difficulties, and heightened parenting stress within the home environment. In this study, we aimed to evaluate the effectiveness of a comprehensive intervention combining motor‐based support for children with pDCD and direct parental support. The primary objective was to determine whether this integrated approach could reduce behavioral and emotional difficulties in children with pDCD and alleviate parenting stress.

## METHODS

### Study design

To evaluate the effectiveness of the intervention, a randomized controlled trial (RCT) following the method described by Curtis et al.^8^ was conducted and reported adhering to the standards outlined in the Consolidated Standards of Reporting Trials (CONSORT) 2010 Statement.^9^ The research complied with the Declaration of Helsinki and received approval from the Ethics Committee of the Graduate School of Comprehensive Rehabilitation, Osaka Metropolitan University (Approval No. 2022‐120; March 13, 2023). Informed consent was obtained from all participants. To minimize assessment bias, blinded evaluators conducted all pre‐ and post‐intervention assessments. Pre‐intervention assessments were conducted approximately 1 week prior to the start of the intervention, and post‐intervention assessments were conducted approximately 1 week after the completion of the 8‐week program for both the intervention and control groups. Randomization was performed using a sealed opaque envelope method. An independent researcher generated the allocation sequence using a computer and placed each assignment into a sequentially numbered, opaque, and sealed envelope. Upon participant enrollment, the next envelope in the sequence was opened to determine group allocation, ensuring concealment. This approach minimized the influence of group‐related expectations on outcome assessments.

### Participant selection criteria

An a priori power analysis was conducted using G*Power 3.1 to determine the required sample size.^10^ Based on the findings of Gao et al., who reported an effect size of 1.00 for a motor‐based intervention targeting DCD, the analysis adopted a large effect size (Cohen's *d* = 1.00), a conventional alpha level of 0.05, and a statistical power of 0.80.^11^ Under these parameters, the calculation indicated that 18 participants (9 per group) would be necessary to detect statistically significant differences using a two‐tailed Mann–Whitney *U* test.

Recruitment was conducted by distributing 82,000 flyers to households in the six municipalities surrounding the university. The flyer stated that the program was intended for children who experience difficulties with physical activity, with the condition that they have no issues with basic communication and do not have cerebral palsy or a rare disease.

In a 2023 report to the Ministry of Health, Labor and Welfare of Japan, Iwanaga et al. noted that formal diagnoses of DCD remain limited, although latent cases are presumed to exist.^12^ Since then, the terms “trait of DCD” or “probable DCD (pDCD)” have often been used in Japan to describe such cases. For example, Ito et al. examined school‐aged children with the trait of DCD,^13^ whereas Nobusako et al. focused on children identified as having pDCD.^14^ In this study, the term pDCD was used to refer to children with DCD. Children were identified as having pDCD if they fulfilled the diagnostic criteria outlined in the Diagnostic and Statistical Manual of Mental Disorders, Fifth Edition, and scored below the 16th percentile on the total score of the Movement Assessment Battery for Children‐Second Edition (MABC‐2), in accordance with the criteria described in the manual by Henderson et al.^15^ Participants with confirmed diagnoses of cerebral palsy or other neuromuscular conditions were excluded. Furthermore, in accordance with the International Clinical Practice Recommendations by Blank et al., which state that a formal diagnosis of DCD under the age of 5 years should only be made in cases of severe impairment due to the large variability in normal motor development, only children aged 6–15 years were deemed eligible for inclusion.^4^

### Baseline characteristics

DCD may co‐occur with ADHD and ASD. These comorbid conditions are associated with psychosocial issues and parenting stress. Therefore, as a general approach, we administered questionnaires to ADHD and ASD.^16^, ^17^

#### ADHD symptom assessment

To assess ADHD symptom severity, we utilized the Japanese version of the ADHD Rating Scale‐IV (ADHD‐RS‐IV), originally developed by DuPaul et al. in 1998 and later supervised by Ichikawa et al. in 2017.^18^, ^19^ This parent‐rated questionnaire comprises 18 items categorized into two subscales: inattention (nine items) and hyperactivity‐impulsivity (nine items). Three scores were calculated: inattention, hyperactivity‐impulsivity, and total score. Raw scores were converted into percentile values based on the child's age and sex, using a standardized scoring sheet. Scores at or above the 93rd percentile on either subscale indicated potential ADHD. The total ADHD‐RS‐IV score was used for analysis in this study.

#### Social Communication Questionnaire

To screen for ASD symptoms, the Japanese version of the Social Communication Questionnaire (SCQ) was used, originally developed by Rutter et al. in 2003 and later adapted under the supervision of Kuroda et al. in 2017.^20^, ^21^ This 40‐item parent‐reported questionnaire assesses ASD‐related behaviors using binary (“yes” or “no”) responses. Two SCQ versions exist: the SCQ‐Current, which evaluates current behavior, and the SCQ‐Lifetime, which addresses developmental history from birth to the present. For this study, the Lifetime version was used for screening. Each item was scored as 0 or 1, and the total score was used to estimate ASD likelihood. According to the Japanese version of the SCQ manual, a cutoff score of ≥15 indicates potential ASD risk.^21^

### Primary outcome measures

#### Movement Assessment Battery for Children‐Second Edition

The MABC‐2 was employed to assess motor skills. This internationally recognized tool is designed to evaluate motor difficulties in children and is structured into three age bands: Band 1 (≤6 years, 11 months), Band 2 (7 years, 0 months to 10 years, 11 months), and Band 3 (≥11 years, 0 months). In this study, participants were assessed using the age band corresponding to their chronological age. Each age group included eight tasks, with raw scores converted to standard scores using age‐specific normative tables. The tasks were grouped into three domains: manual dexterity, aiming and catching, and balance. Domain‐specific standard scores were calculated by summing scores for relevant tasks, and a total standard score was derived by combining the scores across all three domains. Subsequently, these scores were then converted into percentile ranks using normative data.^15^ As the Japanese version of the MABC‐2 remains under development and lacks standardization, score conversion was performed using the original UK norms. Previous studies have demonstrated the strong applicability of these norms to Japanese children aged 7–10 years.^22^ Based on this framework, children scoring below the 16th percentile were classified as having pDCD.

#### Child Behavior Checklist

The Japanese version of the CBCL, originally developed by Achenbach and Rescorla in 2007 and supervised by Funabiki and Murai in 2017, was used to assess children's behavioral, emotional, and social functioning.^23^, ^24^ This parent‐reported questionnaire consists of 118 items describing various problem behaviors, and parents were asked to rate each item on a 3‐point Likert scale: 0 = not true, 1 = somewhat or sometimes true, and 2 = very true or often true. Standardized *T*‐scores were calculated separately by sex. According to Japanese CBCL norms, *T*‐scores < 60 indicate the normal range, scores between 60 and 63 correspond to the borderline range, and scores > 63 indicate the clinical range. In this study, the “Total Problems” score was used as the primary outcome measure.

#### Parenting Stress Index

The Japanese version of the Parenting Stress Index (PSI), originally developed by Abidin in 1995 and supervised by Kanematsu et al. in 2015, was administered specifically to mothers of the target children.^25^, ^26^ The PSI originally consisted of 78 items, with 38 questions regarding the child's perspective and 40 questions regarding the parent's perspective. In this study, only parenting stress items from the child's perspective were adopted to investigate the impact on children with pDCD. Each item was rated on a 5‐point Likert scale ranging from “Not at all true” to “Exactly true,” with respondents instructed to reflect their actual perceptions. Responses were scored using the PSI scoring sheet, with each item assigned a numerical value from 1 to 5. Total scores for the “Child Domain” were calculated by summing the individual item scores. Based on the total score, percentile ranks were identified using the PSI Percentile Table, developed from the normative sample's frequency distribution. Scores falling between the 15th and 80th percentiles were classified as within the standard range, whereas scores at or above the 85th percentile were indicative of elevated parenting stress.

### Intervention group

#### Play‐based intervention for children with pDCD

Participants in the intervention group attended 90‐min physical activity sessions once weekly over an 8‐week period. The program was a task‐oriented motor‐skill training intervention that incorporated game‐based elements (Table 1). Sessions were conducted in a gymnasium by trained assistants under the supervision of a licensed physical therapist with 21 years of experience in pediatric care. In accordance with the international clinical practice recommendations by Blank et al., each session was overseen by one lead facilitator, while an assistant supported approximately four children with pDCD, resulting in a total of three assistants for 10 children.^4^ Prior to the intervention, all assistants completed a 90‐min training session conducted by the researchers. The training addressed the characteristics and associated risks of developmental disorders and outlined the structure and objectives of the motor‐skill training program. Pre‐ and post‐session meetings were held to facilitate information sharing, session reflection, and discussion of areas for improvement. Feedback was delivered to the children in a structured manner; problematic behaviors were addressed clearly and concisely at the beginning of the following session, while positive behaviors were praised immediately. As part of the social skills training, children were reminded at the start of each session to support their peers, refrain from using hurtful language, and offer praise to others.

**Table 1:** Weekly activity schedule (Weeks 1–8).

Weeks	Balance activities (20 min)	Aerobic exercise (30 min)	Ball throwing and catching (20 min)	Fine motor skills (20 min)
1–2 W	Ladder two‐foot jump	Tail tag	Catch ball with balloon	Paper cup tower
	Rock‐paper‐scissors with ladder jump		Ball toss game	Newspaper tearing (as long as possible)
	“Daruma‐san ga Koronda” with two‐foot jump movement			
3–4 W	Ladder hopscotch	Tail tag	Catch ball with volleyball	Dominoes
	Hopscotch rock‐paper‐scissors game		Ball toss game	
	“Daruma‐san ga Koronda” with hopscotch movement			
5–6 W	Ladder hopscotch	Tail tag	Strike‐out game with volleyball	Jenga
	Hopscotch movement rock‐paper‐scissors game		Catch ball with tennis ball	
7–8 W	Ladder one‐leg jump	Tail tag	Catch ball with tennis ball	String figures (broom shape)
	One‐leg jump movement rock‐paper‐scissors game		Strike‐out game with baseball	Origami (paper gun)

#### Parental involvement

Parents were invited to attend the sessions and observe their children's behavior, categorizing it into three types: desirable, underdeveloped, and undesirable. For desirable behaviors, parents provided specific praise, including physical gestures such as patting the child's head or giving a high‐five. In case of underdeveloped behaviors, they were encouraged to acknowledge partial successes and motivate further attempts. For undesirable behaviors, parents approached the child, clearly stated which behavior should stop, and repeated the instruction if necessary. Immediate praise was given if the child ceased the behavior (Table 2).

**Table 2:** Parental support strategies.

Behavior category	Response to desirable behavior	Response to underdeveloped behavior	Response to undesirable behavior
Emotional support	Praise (e.g., pat on the head, high‐five)	Encouragement (acknowledge partial success)	Clear correction and repeated guidance
Social skills	Positive reinforcement (immediate praise)	Guidance (explain the meaning of the behavior)	Instructions to stop the behavior and immediate praise
Learning support	Rewards (e.g., treats)	Assistance (encourage attempts)	Constructive feedback instead of criticism

### Control group

Children in the control group received standard care for pDCD, supported both at school and at home. In schools, physical education classes are routinely adapted by teachers to accommodate individual motor challenges as part of standard instructional practice. Activities such as ball handling, jumping, and balance exercises were modified to promote engagement and a sense of achievement. At home, parents were encouraged to support everyday tasks involving fine motor skills, such as dressing, eating, and handwriting, to strengthen independence and coordination. These widely implemented strategies for children with pDCD in educational and domestic environments are tailored to their developmental stage and functional abilities.

### Statistical analysis

Prior to selecting this test, Shapiro–Wilk tests were conducted to assess the normality of each outcome variable (MABC‐2, CBCL, and PSI ‐C‐hild Domain [PSI‐C]). The results indicated deviations from normality across several measures, thereby justifying the use of nonparametric methods. Given the small sample size (*n* = 10 per group) and the potential for non‐normal distribution of outcome variables, the Mann–Whitney *U* test, a nonparametric alternative to the independent samples *t*‐test, was used to compare changes between the intervention and control groups. Chi‐square tests were used to assess group differences in categorical variables such as sex. Additionally, within‐group pre‐ and post‐intervention comparisons were conducted using the Wilcoxon signed‐rank test. For this test, effect sizes (*r*) and 95% confidence intervals (CIs) were calculated to evaluate the magnitude and precision of the observed effects. A significance level of 0.05 was adopted for all statistical tests. Statistical analyses were conducted using Jamovi software (version 2.6.26 Solid; https://www.jamovi.org).

## RESULTS

### Trial flow

**Figure 1:**
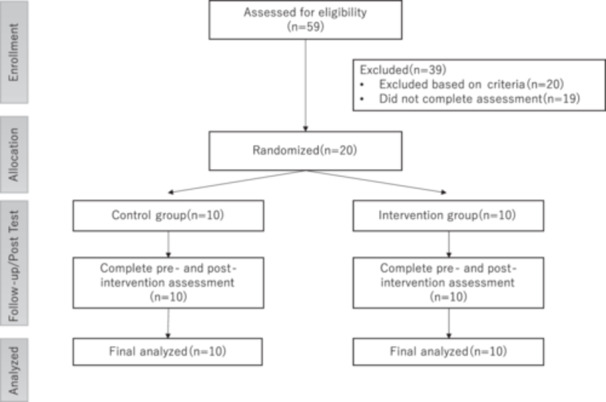
Consolidated Standards of Reporting Trials (CONSORT) diagram depicting participant flow throughout the study.

Figure 1 illustrates the participant flow throughout the study in accordance with CONSORT guidelines. Overall, 59 children were enrolled; of these, 19 did not complete the direct assessment, and 20 were excluded based on the exclusion criteria. The remaining 20 children met the eligibility criteria and were randomly assigned to either the intervention or control group. All participants completed both pre‐ and post‐intervention assessments, with no dropouts during the intervention period (Figure 1). The median attendance rate was 75%, indicating moderate adherence to the intervention program.

### Baseline characteristics

Table 3 presents the baseline characteristics of participants from both the intervention and control groups, including variables such as age, sex, motor skills (assessed through MABC‐2), behavioral problems (evaluated via CBCL), parenting stress (measured through PSI‐C), traits of ADHD (assessed via ADHD ‐RS‐IV), and traits of ASD (measured using SCQ). The median age was 8.0 years for the entire group, as well as for both the intervention and control groups, which was 8.0 years. There were 12 boys and 8 girls in total, with 7 boys and 3 girls in the intervention group and 5 boys and 5 girls in the control group. There were no significant differences between the two groups.

**Table 3:** Baseline characteristics and outcome measures.

	Unit	Control group (IQR)	Intervention group (IQR)	*p* value
Age	(year)	8.0 (2.8)	8.0 (1.8)	0.76
Sex	(male)	7	5	0.68
	(female)	3	5	
MABC‐2	(score)	47.0 (21.0)	56.5 (16.0)	0.19
CBCL	(score)	46.0 (40.5)	42.5 (36.8)	0.79
PSI‐C	(score)	95.0 (31.0)	94.5 (15.5)	0.52
ADHD‐RS‐IV	(%ile)	91.5 (13.5)	93.0 (12.0)	0.47
SCQ	(score)	5.0 (4.5)	5.0 (8.3)	0.68

*Note*: The data for age, MABC‐2, CBCL, PSI‐C, ADHD‐RS‐IV, and SCQ are presented as medians along with interquartile ranges. Sex is reported as the number of individuals. Statistical comparisons of these baseline variables between the two groups were performed using chi‐square tests for categorical variables (sex), and the Mann–Whitney *U* test for continuous and ordinal variables.

Abbreviations: %ile, percentile; ADHD‐RS‐IV, Attention‐Deficit/Hyperactivity Disorder Rating Scale‐Fourth Edition; CBCL, Child Behavior Checklist; IQR, interquartile range; MABC‐2, Movement Assessment Battery for Children‐Second Edition; PSI‐C, Parenting Stress Index‐Child Domain; SCQ, Social Communication Questionnaire.

### Changes in MABC‐2, CBCL, and PSI‐C scores within and between groups

Table 4 summarizes the mean scores and statistical comparisons for the MABC‐2, CBCL Total Problems Scale, and PSI‐C scores, assessed before and after the intervention for both the intervention and control groups.

**Table 4:** Overview of primary outcome measures.

Measure	Group	Pre‐median (IQR)	Post‐median (IQR)	*ｒ*[95% CI]	*p* value
MABC‐2	Intervention	56.5 (16.0)	57.0 (14.0)	−0.36 [−0.81, 0.35]	0.33
	Control	47.0 (21.0)	53.5 (23.5)	−0.6 [−0.89, 0.05]	0.10
CBCL	Intervention	42.5 (36.8)	32.0 (32.8)	0.86 [0.49, 0.97]	0.02*
	Control	46.0 (40.5)	36.5 (26.5)	0.49 [−0.20, 0.86]	0.18
PSI‐C	Intervention	95.0 (31.0)	88.0 (25.8)	0.78 [0.29, 0.95]	0.04*
	Control	94.5 (15.5)	105.5 (28.0)	−0.42 [−0.83, 0.29]	0.28

*Note*: Data are presented as median and interquartile range. Effect size (*r*) was calculated to assess the magnitude of change in each outcome measure within the intervention group and within the control group. Statistical comparisons of pre‐ and post‐continuous variables were performed using the Wilcoxon signed‐rank test.

Abbreviations: CBCL, Child Behavior Checklist; CI, confidence interval; IQR, interquartile range; MABC‐2, Movement Assessment Battery for Children‐Second Edition; PSI‐C, Parenting Stress Index‐Child Domain.

* *p* < 0.05.

The Wilcoxon signed‐rank test indicated no statistically significant differences in the total MABC‐2 scores between the pre‐ and post‐intervention assessments in either group.

For behavioral problems evaluated using the CBCL, no statistically significant change over time was observed in the control group. In contrast, the intervention group exhibited significantly reduced scores following the intervention, suggesting a notable improvement in behavioral outcomes (*p* = 0.02). A larger effect size (*r* = 0.86, 95% CI [0.49, 0.97]) was obtained, indicating a substantial reduction in problem behavior (large effect: *r* ≥ 0.5).

The intervention group showed a statistically significant decrease in the PSI‐C scores after the intervention (*p* = 0.04). A large effect size (*r* = 0.78, 95% CI = [0.29, 0.95]) was observed in the intervention group. In contrast, no statistically significant changes were observed over time in the control group.

### Between‐group comparisons of post‐intervention outcomes

Between‐group comparisons of the post‐intervention scores were conducted for the primary outcomes. For motor skills (MABC‐2), the Mann–Whitney *U* test indicated no significant difference between the intervention and control groups (*p* = 0.45), with a small effect size (*r* = 0.21). No significant difference was found in behavioral problems (CBCL) (*p* = 0.94, *r* = −0.03). For parenting stress (PSI‐C), the difference was not significant (*p* = 0.34), with a small effect size (*r* = 0.26).

## DISCUSSION

Based on existing literature, this is the first RCT conducted in Japan to evaluate the effectiveness of a comprehensive intervention combining motor training and direct parental support for children with pDCD. The primary aims were to improve motor skills, reduce behavioral and emotional difficulties, and alleviate parenting stress.

No significant changes were observed in motor performance (MABC‐2) in either the intervention or control group. However, behavioral problems significantly decreased in the intervention group, with a large effect size (*r* = 0.86). Parenting stress in the Child domain also decreased significantly, and the effect size was large (*r* = 0.78). These findings suggest that a comprehensive intervention targeting both children and their parents can effectively reduce behavioral problems, even in the absence of measurable improvements in motor skills, and may help alleviate parenting stress in the Child domain. Although within‐group improvements were observed in behavioral problems and parenting stress in the Child domain, between‐group comparisons of post‐intervention scores did not reach statistical significance for any primary outcome. The small effect sizes for MABC‐2 (*r* = 0.21), CBCL (*r* = −0.03), and PSI‐C (*r* = 0.26) suggest that while trends may favor the intervention group, the magnitude of change was limited in this pilot study.

### Reasons for lack of motor skill improvement

In this study, no statistically significant improvement was observed in motor skills, as assessed using the MABC‐2. One possible explanation is the relatively short duration of the intervention (8 weeks), which may have limited opportunities for skill acquisition and consolidation. Another possible factor is the difference in contact time with professionals between the previous studies and the present study. Improvements may have been influenced not only by the content of the program but also by increased interaction with specialists, which could have acted as a confounding variable.^11^ Gao et al.^11^ reported that the effects may be limited in the short term (6–8 weeks), and interventions are primarily provided by physical therapists, occupational therapists, educators, and researchers, with parents or teachers assisting with interventions occurring only in some cases.

Previous research has emphasized the importance of parental involvement and environmental support in promoting motor development. Steenbergen et al.^27^ highlighted that social networks and sustained engagement are critical in encouraging participation in children with DCD. Similarly, Rosenblum and Engel‐Yeger^28^ emphasized the role of parents as mediators of participation and the need for environmental adjustments in school settings. These findings suggest that a longer intervention period and more intensive parental involvement are necessary to achieve measurable improvements in motor skills.

### Improved behavioral outcomes

Although motor skills did not significantly improve in this study, behavioral challenges were notably reduced in the intervention group. This positive outcome may be attributed to the intervention's emphasis on promoting social participation, enhancing social skills through training, and fostering prosocial behavior via parent‐focused support. In their systematic review and meta‐analysis, Gao et al. suggested that motor‐based interventions for children with DCD may have potential benefits for psychosocial outcomes.^11^ Darling et al. found that behavioral interventions significantly improved social functioning and cognition in children with neurodevelopmental disorders, supporting the role of social skills training in broader behavioral outcomes.^29^ Children with pDCD often experience social withdrawal due to motor‐related anxiety or peer rejection.^27^ Participation in group‐based physical activities and cooperative games in this study offered children with pDCD a supportive environment to connect with peers, potentially enhancing their sense of belonging and social competence. This structured social exposure likely played a key role in reducing behavioral problems, independent of motor skill improvements. The implementation of a parent training program may have contributed to a reduction in the frequency of behavioral problems by enhancing caregivers' consistency and responsiveness in the home environment. Through structured guidance, parents appeared to acquire more effective strategies for managing challenging behaviors, which likely improved the overall quality of parent–child interactions. Furthermore, the use of positive feedback, emphasizing strengths rather than deficits, may have reinforced desirable behaviors and diminished problematic ones. This approach aligns with behaviorist principles, suggesting that the consistent reinforcement of adaptive behaviors can lead to meaningful behavioral changes.

### Reduction in parenting stress

In our previous study, some parents reported reduced parenting stress following improvements in their children's motor skills, while others experienced increased stress despite these advancements.^30^ This variability suggests that parenting stress may be influenced by factors beyond motor performance. Bloomfield's study involving 63 parents of children under 10 years old revealed a strong negative correlation between parenting self‐efficacy and stress, but only a weak correlation with child behavior.^31^ In this study, parenting stress was addressed by encouraging the use of positive rather than negative language when providing feedback. This practice may have contributed to improved parent–child relationships and, consequently, enhanced parenting self‐efficacy. The parent training conducted in this study may have influenced parenting self‐efficacy, which, in turn, likely led to reduced parenting stress.

### Clinical implications

The multidimensional approach evaluated in this study has the potential to enhance children's social participation and decrease parenting stress, thereby fostering a more inclusive and nurturing developmental environment. Our findings suggest that, even without significant improvements in motor skills, enhancements in social competencies and parent–child relationships may help alleviate behavioral and emotional difficulties in children with pDCD, as well as parenting stress.

Overall, the results support the viability of an integrated support program encompassing physical activity, social participation, social skills development, and parental training as an effective intervention strategy for children with pDCD and their families. Furthermore, this study presents a culturally relevant intervention model that may inform global efforts to support children with DCD, particularly in countries where formal diagnostic and support systems are emerging. By demonstrating both the feasibility and effectiveness of a multidimensional approach within a Japanese context, these findings contribute to the global discourse on inclusive, family‐centered care for neurodevelopmental disorders.

### Study limitations

A limitation of this study is the lack of blinding among parents who were aware of their children's group assignment. This may have introduced reporting bias in parent‐rated measures, such as the CBCL and PSI‐C. Although an RCT design was employed, the relatively small sample size limited the statistical power to detect the subtle effects. In addition, information regarding the participants' current or past pharmacological treatment, formal clinical diagnoses (e.g., ADHD, ASD), and intellectual functioning (e.g., intelligence quotient scores) was not collected in this study. Comorbid traits were assessed only using screening tools, and future studies should further examine their potential influence on intervention outcomes. Furthermore, owing to the small number of participants, the study results cannot be generalized, underscoring the need for larger sample sizes and longer intervention durations to detect meaningful between‐group differences in future studies.

## AUTHOR CONTRIBUTIONS

Ryota Hatanaka contributed to the conception and design of the study, data acquisition, analysis, interpretation, and drafted the manuscript. Yasuko Takahashi and Ayako Hisari contributed to data acquisition and writing—review and editing. Keiko Sakai contributed to resources and provided substantial input in writing—review and editing. Yumi Higuchi and Masatoshi Takeda contributed to supervision and writing—review and editing. All authors have read and agreed to the published version of the manuscript.

## CONFLICT OF INTEREST STATEMENT

The authors declare no conflicts of interest.

## ETHICS APPROVAL STATEMENT

The research was conducted in accordance with the Declaration of Helsinki, and the protocol was approved by the Ethics Committee of the Graduate School of Comprehensive Rehabilitation, Osaka Metropolitan University (Approval No. 2022‐120, dated March 13, 2023).

## PATIENT CONSENT STATEMENT

Informed consent was obtained from all participants (100% consent rate).

## CLINICAL TRIAL REGISTRATION

Due to an administrative oversight, the study was retrospectively registered with the University Hospital Medical Information Network Clinical Trials Registry (UMIN000058187) on June 16, 2025, following the completion of participant enrollment (2023–2024). All study procedures were approved by the institutional ethics committee prior to initiation and conducted in accordance with the Declaration of Helsinki.

## Supporting information

Supporting Information.

## Data Availability

Data that support the findings of this study are available in the Supporting Information of this article. The data utilized in this study are not publicly accessible due to privacy protection and ethical considerations. However, they may be made available from the corresponding author upon reasonable request.
