# Supplementary material for: Impact of play‐based intervention and parental support on motor skills, behavioral problems, and parenting stress in Japanese children with probable developmental coordination disorder: A randomized controlled trial
Source: PCN Rep. 2025 Nov 24;4(4):e70256. doi: 10.1002/pcn5.70256 (PMC12643948; doi:10.1002/pcn5.70256)
Supplement: Supplementary file 1 — Supporting Information. [file PCN5-4-e70256-s001.docx]

# CONSORT-NPT Checklist (Filled Based on Manuscript)

| Item No. | Checklist Item | Reported Status | Comments |
| --- | --- | --- | --- |
| 1a | Identification as a randomized trial in the title | Reported | Clearly described in manuscript |
| 1b | Structured summary of trial design, methods, results, and conclusions | Reported | Clearly described in manuscript |
| 2a | Scientific background and explanation of rationale | Reported | Clearly described in manuscript |
| 2b | Specific objectives or hypotheses | Partially reported | Some elements present; consider elaborating |
| 3a | Description of trial design (such as parallel, factorial) including allocation ratio | Reported | Clearly described in manuscript |
| 3b | Important changes to methods after trial commencement (such as eligibility criteria), with reasons | Reported | Clearly described in manuscript |
| 4a | Eligibility criteria for participants | Reported | Clearly described in manuscript |
| 4b | Settings and locations where the data were collected | Reported | Clearly described in manuscript |
| 4c | Details of care providers, centers, and settings | Reported | Clearly described in manuscript |
| 5a | The interventions for each group with sufficient details to allow replication | Reported | Clearly described in manuscript |
| 5b | Description of how interventions were standardized | Reported | Clearly described in manuscript |
| 5c | Details of how adherence to the intervention was assessed or enhanced | Reported | Clearly described in manuscript |
| 6a | Completely defined pre-specified primary and secondary outcome measures | Reported | Clearly described in manuscript |
| 6b | Any changes to trial outcomes after the trial commenced, with reasons | Reported | Clearly described in manuscript |
| 7a | How sample size was determined | Reported | Clearly described in manuscript |
| 7b | When applicable, explanation of any interim analyses and stopping guidelines | Reported | Clearly described in manuscript |
| 8a | Method used to generate the random allocation sequence | Reported | Clearly described in manuscript |
| 8b | Type of randomization; details of any restriction (such as blocking and block size) | Reported | Clearly described in manuscript |
| 8c | Mechanism used to implement the random allocation sequence | Reported | Clearly described in manuscript |
| 9 | Who generated the random allocation sequence, who enrolled participants, and who assigned participants to interventions | Reported | Clearly described in manuscript |
| 10 | Implementation of blinding (masking) | Partially reported | Some elements present; consider elaborating |
| 11a | If done, who was blinded after assignment to interventions and how | Reported | Clearly described in manuscript |
| 11b | If relevant, description of the similarity of interventions | Reported | Clearly described in manuscript |
| 11c | Blinding of care providers and participants | Reported | Clearly described in manuscript |
| 12a | Statistical methods used to compare groups for primary and secondary outcomes | Reported | Clearly described in manuscript |
| 12b | Methods for additional analyses, such as subgroup analyses and adjusted analyses | Reported | Clearly described in manuscript |
| 13a | Participant flow (a diagram is strongly recommended) | Reported | Clearly described in manuscript |
| 13b | For each group, the numbers of participants who were randomly assigned, received intended treatment, and were analyzed for the primary outcome | Reported | Clearly described in manuscript |
| 13c | Losses and exclusions after randomization, together with reasons | Reported | Clearly described in manuscript |
| 14a | Dates defining the periods of recruitment and follow-up | Reported | Clearly described in manuscript |
| 14b | Why the trial ended or was stopped | Reported | Clearly described in manuscript |
| 15 | Baseline demographic and clinical characteristics for each group | Reported | Clearly described in manuscript |
| 16 | Number of participants (denominator) included in each analysis | Reported | Clearly described in manuscript |
| 17a | For each primary and secondary outcome, results for each group, and the estimated effect size and its precision | Reported | Clearly described in manuscript |
| 17b | For binary outcomes, presentation of both absolute and relative effect sizes is recommended | Reported | Clearly described in manuscript |
| 18 | Results of any other analyses performed, including subgroup analyses and adjusted analyses | Reported | Clearly described in manuscript |
| 19 | All important harms or unintended effects in each group | Reported | Clearly described in manuscript |
| 20 | Trial limitations, addressing sources of potential bias, imprecision, and, if relevant, multiplicity of analyses | Reported | Clearly described in manuscript |
| 21a | Generalizability (external validity, applicability) of the trial findings | Reported | Clearly described in manuscript |
| 21b | Feasibility and implementation of the intervention | Reported | Clearly described in manuscript |
| 22 | Interpretation consistent with results, balancing benefits and harms, and considering other relevant evidence | Reported | Clearly described in manuscript |
| 23 | Registration number and name of trial registry | Reported | Clearly described in manuscript |
| 24 | Where the full trial protocol can be accessed, if available | Reported | Clearly described in manuscript |
| 25 | Sources of funding and other support; role of funders | Reported | Clearly described in manuscript |
